# Double plating for long-standing nonunion of the humeral shaft complicated with metaphyseal bone defect and deformity: A case report

**DOI:** 10.1016/j.tcr.2021.100448

**Published:** 2021-03-01

**Authors:** Natsumi Saka, Gen Sasaki, Yoshinobu Watanabe, Hirotaka Kawano

**Affiliations:** Department of Orthopaedics, Teikyo University School of Medicine, Tokyo, Japan

**Keywords:** Nonunion, Metaphyseal, Autograft, Elbow, Humerus

## Abstract

Several treatment methods for nonunion of humeral fracture have been reported with satisfactory results. However, treatment of a long-standing nonunion of the humerus is more challenging, as it may be complicated by broken implants and bone defects. Little is known about treatment strategies for long-standing humeral nonunion with bone defects, especially in the metaphyseal area. We report a case of long-standing humeral shaft nonunion complicated by a bone defect and deformity, treated with double locking plates and an iliac bone autograft.

## Introduction

It is reported that humeral shaft nonunion occurs in 3% of all surgically treated fractures [1]. Instability in the nonunion leads to pain and difficulty in the involved upper extremity during heavy lifting.

Several treatment methods have been reported, including fixation by intramedullary nailing, plating, augmentation plating after nailing, and external fixation [[2], [3], [4], [5]]. Regarding osteogenic and osteoconductive factors, some studies describe simple additional fixation, while others report the use of autografts, vascularized bone grafts, and allografts [3,5]. The overall reported union rates in those surgeries are favorable [2,[4], [5], [6]].

For a long-standing humeral shaft nonunion compared to a recently diagnosed nonunion, treatment is more challenging, because it can be complicated with a larger bone defect and implant breakage [7]. However, little is known about treatment of this pathology. Here, we describe a case of long-standing humeral shaft nonunion complicated with a bone defect and deformity treated with double locking plates and iliac bone autograft.

## Case report

**Fig. 1:**
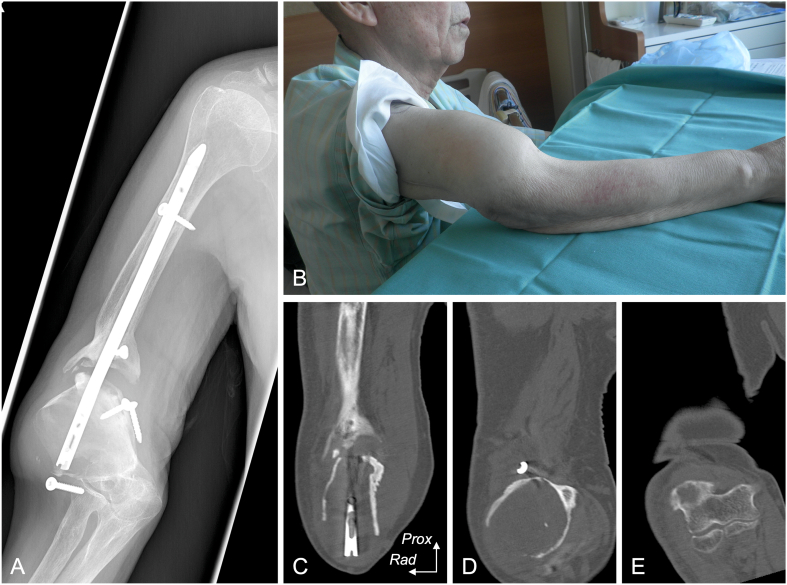
Preoperative findings. A) X-ray revealed nonunion and deformity of the distal fragment. B) Physical findings showed the deformity in the upper arm. C, D) Coronal section of CT showed a bone defect and cavity formation in the distal metaphyseal area. E) The joint surface of the elbow remained intact. *Prox*, *proximal*, *Rad*, *radial*.

The patient was a 78-year-old male referred to our hospital for the treatment of humeral nonunion with worsening pain in the right upper arm. He had a history of hypertension, hyperlipidemia, atrial fibrillation, and aortic valve replacement due to the aortic stenosis. Twenty years ago, he sustained the right humeral shaft fracture in a motor vehicle accident. He underwent internal fixation by retrograde intramedullary nailing, which failed to unite. On physical finding, there was a deformity on the upper arm (Fig. 1B). Shoulder movement was restricted completely and the active range of motion (ROM) of the right elbow was −40° of extension and 60° of flexion. The Disabilities of the Arm, Shoulder and Hand (DASH) score was 83.9. X-rays and computed tomography (CT) showed the nonunion with a bone defect at the humeral shaft, as well as a bone cavity with ballooning deformity at the distal metaphyseal area (Fig. 1A, C, D). Preoperative blood examination revealed white blood cell counts of 6200 cells/mm^3^ and C-reactive protein of 0.1 mg/L. The patient underwent reconstruction of the nonunion four months after the presentation. While waiting for the surgery, he developed radial nerve palsy due to instability of the nonunion, resulting in the inability to extend the wrist and fingers.

## Surgical technique

**Fig. 2:**
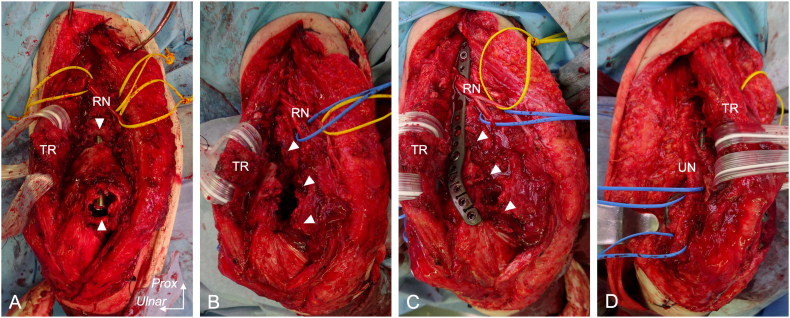
Intraoperative findings. A) After debridement of fibrous tissue and sclerotic bone, the bone defect and distal end of the nail was observed (white arrow). B) After removal of the nail, the bone defect was observed from the radial side (white arrow). C) Bone defect was filled with iliac bone autograft after fixation by the locking plate (white arrow). D) After dissection of the ulnar nerve, the ulnar side was fixed by the locking plate. *Prox*, *proximal*, *RN*, *radial nerve*; *UN*; *ulnar nerve TR*; *triceps*.

**Fig. 3:**
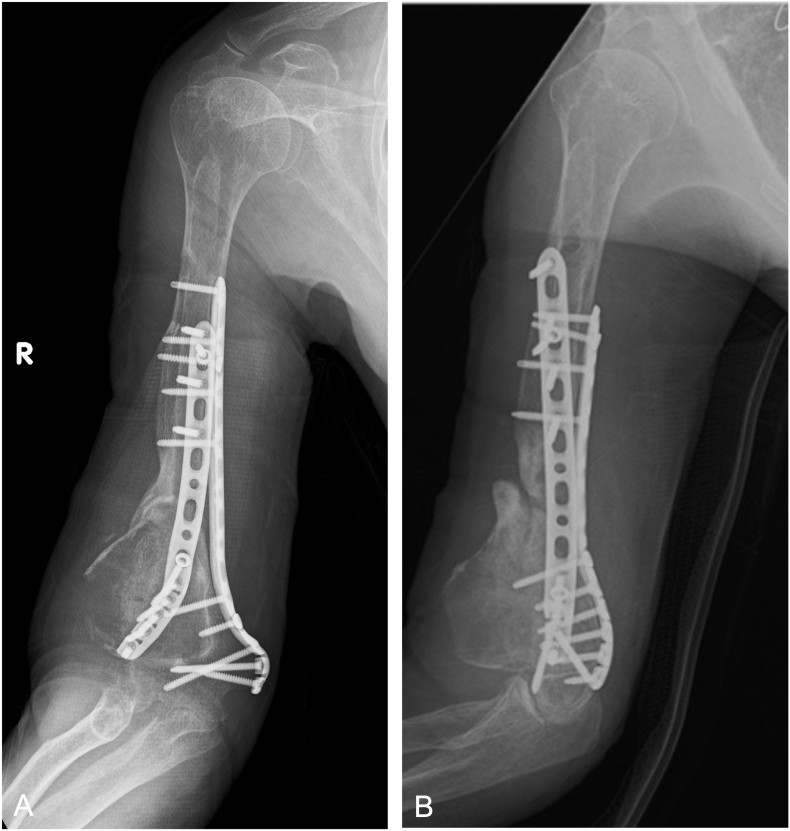
Postoperative X-ray findings. A) Anterior posterior view of the humerus. B) Lateral view of the humerus.

Surgery was performed under general anesthesia in the prone position, using a posterior approach. After retracting the triceps, the radial and ulnar nerves were isolated and protected. Subsequently, fibrous tissue as well as sclerotic bone that had developed around the distal fragment were excised, and the nail and screw were removed (Fig. 2A, B). A minute amount of serous fluid was detected in the bone defect; on sending it for culture examination, the result obtained was negative. Double plating from the posterolateral and ulnar side was performed using an anatomically contoured implant (A.L.P.S.® Elbow Fracture System; Biomet Orthopedics, US) (Fig. 2C, D). The plate was placed using the ulnar cortex as a guide, leaving 7-cm long and 2-cm wide bone defect at the radial side and 3 cm × 3 cm × 2 cm of bone cavity at the distal fragment. We harvested the corticocancellous bone block (5 cm × 2 cm × 2 cm) as well as cancellous bone chips from posterior iliac crest. The block was used to reconstruct the radial side bone defect and cancellous bone chips was used to fill the defect at the ulnar side and cavity (Fig. 2C). After irrigation, the incision was closed. Fig. 3A, B shows postoperative x-rays.

## Postoperative course

**Fig. 4:**
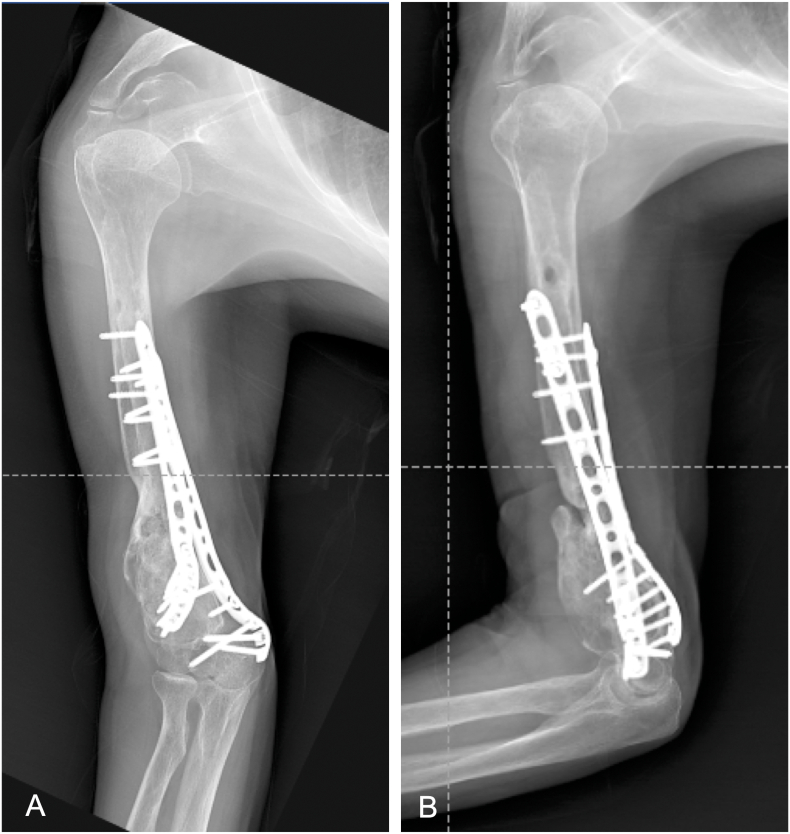
X-ray findings one year after the surgery. A) Anterior posterior view of the humerus. B) Lateral view of the humerus.

After surgery, a protective splint was applied for several days and ROM exercise was initiated. Postoperative course was uneventful. One year postoperatively, the patient exhibited complete radial nerve palsy recovery, and x-rays showed consolidation of the nonunion (Fig. 4A, B). Active ROM of the right elbow was −20° of extension and 90° of flexion. Active ROM of the right shoulder was 90° of abduction and 90° of flexion. Postoperative DASH score was 28.6.

## Discussion

In addition to the nonunion at the original fracture site, this case was characterized by a bone defect and deformity of the distal fragment caused by the windshield-wiper effect of the intramedullary nailing. There were three crucial issues in the treatment of this pathology.

One of the issues was the fixation method. For the treatment of humeral nonunion, several fixation techniques have been proposed, including intramedullary nailing, plating, and external fixation. In a case of long-standing nonunion, the use of intramedullary nailing combined with bone autograft was reported by De Carolis in 2018 [5]. Although nailing provides stability to the fracture without jeopardizing soft tissue, it was challenging to perform intramedullary nailing in this case due to the low bone stock in the distal fragment. External fixation is another choice, but complications may include pin site infection, soft tissue contracture, as well as discomfort for the patient. Here, we used a double locking plate. Biomechanically, double plating compared to single plating has exhibited more rigid fixation [8]. In addition, an anatomical locking plate enabled stable fixation, even in a distal fragment with low bone quality. One downside of double plating is the risk of nerve injury. The reported case had preoperative radial nerve palsy. However, meticulous surgical dissection prevented exacerbation of the palsy, and the patient showed recovery during the postoperative course.

Another issue was bone defect in the distal fragment as well as nonunion. Shortening of limb length can be a choice in the upper arm, and this method has no donor site morbidity for the bone graft. However, in our case, there was a need to fill the bone cavity in the distal fragment, and simple shortening could not be performed. For filling a bone defect, iliac autograft has been the standard of care, but other methods such as allograft, autograft by reamer irrigator aspirator (RIA) have also been reported. The quality of the bone graft is generally poor in the elderly and we need to be prepared for the situation wherein sufficient amount of autograft cannot be obtained. RIA allows the surgeon to harvest large amount of autologous bone graft and De Carolis et al. described the use of intramedullary nailing combined with bone graft harvesting by RIA in long-standing nonunion of the humerus [5]. However, there is a risk of complications in RIA, such as femur fractures and bleeding. Mixture of autologous cancellous bone from the iliac crest with osteoconductive material, such as beta-tricalcium phosphate (β-TCP), is also reported [9]. We prepared β-TCP in this case, but used only iliac bone autograft, as the amount and quality of the bone graft was sufficient. As a result, it functioned as an excellent osteogenic and osteoconductive factor for bone union and consolidation of the bone cavity.

Finally, restoration of the elbow joint function can be problematic, especially in nonunion involving the distal humerus. In the treatment of nonunion of the humerus when the distal bone fragment is limited, several studies have reported recovery of elbow function by total elbow arthroplasty [10]. We planned the anatomical reconstruction of the elbow using an anatomically designed locking plate, as the joint surface of the elbow remained intact. After surgery, there was a remaining anterior ballooning deformity in the metaphyseal area, but sufficient elbow function was achieved.

## Conclusion

In summary, we successfully treated a long-standing nonunion complicated with a bone defect and deformity using a double locking plate and iliac bone autograft. Rigid and anatomical fixation with adequate supply of the autograft was the critical factor in the successful treatment of this injury.

## CRediT authorship contribution statement

N.S.: acquisition of data, interpretation of data, and drafting of the manuscript.

G.S.: acquisition of data, critical revision of the manuscript.

Y.W.: acquisition of data, interpretation of data, critical revision of the manuscript and supervising the work.

H.K.: critical revision of the manuscript and supervising the work.

## Consent

Written informed consent was obtained from the patient for the publication.

## Declaration of competing interest

The authors declare that they have no competing interests.

## References

[1] Rommens P.M., Kuechle R., Bord T. (2008). Humeral nailing revisited. Injury.

[2] Lavini F, Renzi Brivio L, Pizzoli A, Giotakis N, Bartolozzi P. Treatment of non-union of the humerus using the Orthofix external fixator. Injury 2001;32:Sd35–40. 10.1016/s0020-1383(01)00114-0 11812477

[3] Van Houwelingen A.P., McKee M.D. (2005). Treatment of osteopenic humeral shaft nonunion with compression plating, humeral cortical allograft struts, and bone grafting. J. Orthop. Trauma.

[4] Allende C., Vanoli F., Gentile L., Gutierrez N. (2018). Minimally invasive plate osteosynthesis in humerus nonunion after intramedullary nailing. Int. Orthop..

[5] De Carolis O, Mori CM, Vicenti G, et al. A lifelong story: case report of a humeral shaft nonunion successfully treated after 30 years. Injury 2018;49:S43-. 10.1016/j.injury.2018.11.031 30526949

[6] Miska M, Findeisen S, Tanner M, et al. Treatment of nonunions in fractures of the humeral shaft according to the Diamond Concept. Bone Joint J 2016;98:81-. 10.1302/0301-620X.98B1.35682 26733519

[7] Feng D., Zhang J., Zhu Y. (2018). Plate fixation with autogenous bone grafting for longstanding humeral shaft nonunion: a retrospective study of 6 cases. Medicine (Baltimore).

[8] Rubel I.F., Kloen P., Campbell D. (2002). Open reduction and internal fixation of humeral nonunions: a biomechanical and clinical study. J. Bone Joint Surg. Am..

[9] Sasaki G., Watanabe Y., Miyamoto W. (2018). Induced membrane technique using beta-tricalcium phosphate for reconstruction of femoral and tibial segmental bone loss due to infection: technical tips and preliminary clinical results. Int. Orthop..

[10] Cil A., Veillette C.J., Sanchez-Sotelo J., Morrey B.F. (2008). Linked elbow replacement: a salvage procedure for distal humeral nonunion. J. Bone Joint Surg. Am..

